# Cost-Effective PCR-Based Identification of *Tunga penetrans* (Siphonaptera) Larvae Extracted from Soil Samples Containing PCR Inhibitor-Rich Material

**DOI:** 10.3390/insects14010005

**Published:** 2022-12-21

**Authors:** Billy L. Amugune, Abneel K. Matharu, Paul Ouma, Francis Mutebi, Lynne Elson, Ulrike Fillinger, Jürgen Krücken

**Affiliations:** 1 International Centre of Insect Physiology and Ecology, Human Health Theme, Nairobi 00100, Kenya; 2 Institute for Parasitology and Tropical Veterinary Medicine, Freie Universität Berlin, 14163 Berlin, Germany; 3 School of Veterinary Medicine and Animal Resources, College of Veterinary Medicine, Animal Resources and Biosecurity, Makerere University, Kampala 10218, Uganda; 4 KEMRI-Wellcome Trust Research Programme, Kilifi 80108, Kenya; 5 Centre for Tropical Medicine and Global Health, Nuffield Department of Medicine, University of Oxford, Oxford OX3 7BN, UK

**Keywords:** Tungiasis, *Tunga penetrans*, molecular entomology, DNA isolation, Phusion^®^ polymerase, FIREpol^®^ Taq polymerase, low-cost PCR

## Abstract

**Simple Summary:**

*Tunga penetrans* is an invasive flea that causes tungiasis—a neglected skin parasitosis—in humans and animals. All juvenile *T. penetrans* stages (eggs, larvae; pupa) are found in sandy soil, while adults survive on their hosts, with females penetrating the skin to breed. Morphological identification of adult fleas is possible, but due to the absence of a morphological key for the juvenile stages, it is currently impossible to conclusively identify these stages of the flea that use morphological features. To understand the ecology of *T. penetrans,* it is important to identify where the off-host development of fleas occurs by sampling soil for monitoring and surveillance studies. For this purpose, a low-cost PCR-based tool for the identification of *T. penetrans* is desirable, since the flea is endemic, predominantly in low-income regions. Since flea larvae feed on organic material in the soil, which is known to be rich in PCR inhibitors, this is rather challenging. We tested six protocol combinations based on three DNA preparation methods and two PCR enzymes to determine the most efficient and economical protocol. The developed protocols can be used in future studies and reduce the costs by more than 80%, when compared with more conventional approaches.

**Abstract:**

Tungiasis is a neglected tropical disease caused by skin-penetrating female *Tunga penetrans* fleas. Although tungiasis causes severe health problems, its ecology is poorly understood and morphological descriptions of the larvae are unavailable. To identify *T. penetrans* immature stages and sites where they develop, diagnostic PCRs are required. However, flea larvae feed on soil organic matter rich in PCR inhibitors. Here, three DNA preparation methods, including a soil DNA kit that removes inhibitors, a simple ammonium acetate precipitation approach (AmAcet) and a crude lysate of larvae (CL), were combined with amplification by the highly processive FIREPol^®^ Taq or the inhibitor-resistant Phusion^®^ polymerase. Independent of the polymerase used, the frequency of successful amplification, C_q_ values and PCR efficacies for the low-cost CL and AmAcet methods were superior to the commercial kit for amplification of a 278 bp partial internal transcribed spacer-2 (ITS-2) and a 730 bp pan-Siphonaptera cytochrome oxidase II PCR. For the CL method combined with Phusion^®^ polymerase, the costs were approximately 20-fold lower than for the methods based on the soil DNA kit, which is a considerable advantage in resource-poor settings. The ITS-2 PCR did not amplify *Ctenocephalides felis* genomic or *Tunga trimammilata* ITS-2 plasmid DNA, meaning it can be used to specifically identify *T. penetrans*.

## 1. Introduction

Tungiasis is a neglected tropical skin disease that affects humans [1] and domestic animals, such as pigs, dogs, and cats [2], as well as wildlife [3]. Although the disease is often considered to be only a nuisance and is, therefore, extremely neglected [4], human disease can be very severe without treatment [5,6]. Domestic animals can also show severe pathology [7,8,9,10]. For example, pigs can be infected by several hundred fleas at the same time [9], which can be associated with severe pathology.

Fleas (order: Siphonaptera) are blood-feeding parasitic arthropods with holometabolic development [11]. Although adults of all flea species are parasitic, the vast majority of a flea population consists of non-parasitic off-host stages in the environment, i.e., eggs, developing larvae and pupae [12]. Larvae feed on organic matter in the host dwellings or the soil and many species also feed on blood-rich flea feces or prey on other arthropods, including other flea larvae [13].

Host specificity of many flea species is low, but there are also many species that are highly adapted to one or a small number of host species [14] and many species at least transiently feed on humans [11]. The most important synanthropic flea species include the human flea *Pulex irritrans* (worldwide), which also infests many animal species, the cat and dog fleas *Ctenocephalides felis* and *Ctenocephalides canis* (worldwide), the rodent fleas *Xenopsylla cheopis* (in the tropics and subtropics) and *Nosopsyllus fasciatus* (in moderate climatic regions), the sticktight flea *Echidnophaga gallinacea* (tropical and subtropical areas) and the sand fleas *Tunga penetrans* and *Tunga trimamillata* [11,15], among others. Thus, the off-host stages of a wide range of flea species can be expected to be present in the environment.

Therefore, investigation of flea ecology has to focus not only on the availability of hosts, but also on suitable sites for off-host development of juvenile stages [16,17,18]. Among the important environmental parameters, temperature and humidity profiles, available feed for larvae and availability of hosts for the next generation of imagines are the most important. However, currently, no estimates about the optima for any of the obvious parameters for off-host development are available for *T. penetrans*. In addition, no systematic studies on the spatial or seasonal occurrence of the off-host stages of *T. penetrans* have been reported.

For flea control, detailed ecological and physiological knowledge is important in order to develop approaches that interfere with the development of off-host stages. Targeting off-host stages has the obvious advantage that the exposure of humans and domestic animals to drugs can be minimized. In order to optimize strategies that target the off-host stages for tungiasis control, it is important to have detailed information about their localization to also minimize side effects on non-target arthropods. The fact that many commercialized drug combinations that target fleas of companion animals also target off-host stages emphasizes their importance in the flea developmental cycles and validates them as a suitable target for intervention [19].

In consideration of its importance for human health [4], the knowledge about ecology of off-host stages in Africa is very scarce. Therefore, more studies are needed to generate better information for the informed implementation of public health strategies. *Tunga penetrans* was brought by humans to Western Africa (Angola) in the 19th century and it rapidly dispersed to Eastern Africa and to Madagascar, with the movements of colonial military troops contributing substantially to this geographical range expansion [20]. Although its expansion into and throughout the African continent is largely human-driven, the local ecological conditions must support the developmental cycle of the flea. Prevalence of *T. penetrans* in Africa can significantly vary even between sites in close geographical proximity [21]. Assuming that the number of available hosts is not limited in human settlements, it is most likely that differences in the environmental conditions required to support off-host stage development contribute to the observed differences in the abundance of *T. penetrans*.

In order to study the off-host ecology of *T. penetrans* and identify development sites for interventions, there is a need to identify the species of the flea larvae with certainty. In the absence of valid morphological identification keys for off-host stages, molecular diagnostics are useful in the identification of flea larvae and other off-host flea stages collected from various microenvironments, since DNA sequence information obtained from adult fleas can be used as a reference. Morphological features from specimens identified by molecular techniques can contribute to the generation of morphological keys for *T. penetrans* juvenile stages. However, morphological identification requires experienced personnel and may be prone to errors. Whilst molecular techniques require robust infrastructure, they are very specific and can, thus, support identifications based on morphology. To increase the utility of molecular methods in low-income countries, the protocols should be as cost-effective as possible.

PCR inhibitors, such as those found in the soil, may limit the utility of molecular diagnostics. In particular, soil, as the habitat from which flea larvae are collected, is well known to contain high concentrations of PCR inhibitors [22,23,24]. There are DNA isolation kits available that are able to remove such inhibitors during DNA isolation and also DNA polymerases that are less susceptible to the effects of inhibitors [25,26,27,28]. However, the use of such kits and polymerases further increases the costs for epidemiological surveys. The aim of the present study was to compare different combinations of methods for DNA isolation and PCR of flea larvae collected from human dwellings to achieve robust amplification of target sequences.

## 2. Materials and Methods

### 2.1. Experimental Design

**Figure 1:**
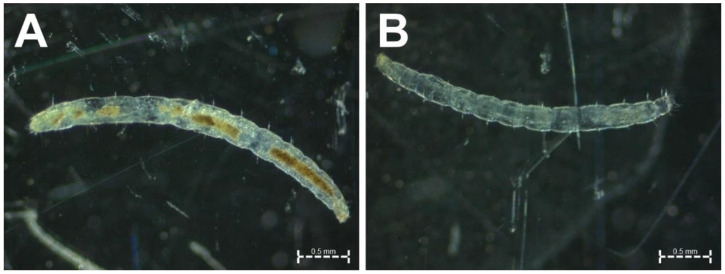
Images of flea larvae taken under a stereo microscope. Presence of soil or organic matter in the flea gut was observed in some larvae (**A**), but not all (**B**). The scale bars represent 0.5 mm.

**Figure 2:**
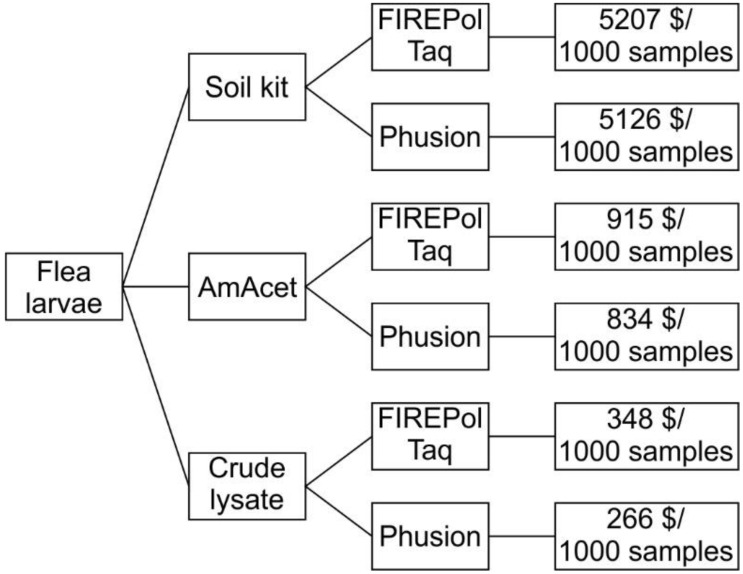
DNA preparation and PCR methods evaluated. Three methods were used to obtain DNA from flea larvae, including a soil DNA isolation kit (S-kit), an ammonium acetate precipitation protocol (AmAcet), and a crude flea lysate protocol. Samples from all extraction protocols were used for PCR amplification using either a hot-start FIREPol^®^ *Taq* DNA polymerase or the highly inhibitor-resistant Phusion^®^ HF DNA polymerase.

Different combinations of DNA extraction protocols and PCR amplification enzymes were evaluated to identify an efficient, low-cost identification tool for *T. penetrans* larvae collected from soil samples taken from potential flea development sites. The high content of PCR inhibitors provides a challenge for PCR assays that target *T. penetrans* larvae, due to the evident presence of soil and organic matter in their gut (Figure 1). In the present study, the following three DNA isolation protocols were used: (1) a low-cost DNA preparation protocol using ammonium acetate; (2) a crude flea lysate (CL) protocol (both developed in this study) and (3) a protocol using the NucleoSpin^®^ Soil DNA isolation kit with the removal of inhibitors (Macherey-Nagel, Düren, Germany) as a standard for comparison. In an initial preliminary comparison, conventional Taq polymerase and the highly inhibitor-resistant Phusion^®^ DNA polymerase (New England Biolabs, Ipswich, MA, USA) were used. Phusion^®^ DNA polymerase is a thermostable polymerase with high proofreading activity fused to a protein domain-binding double-stranded DNA. Due to its poor performance, conventional Taq polymerase was not further evaluated and was replaced by FIREPol^®^ Taq (Solis BioDyne, Tartu, Estonia), a modified Taq with an unchanged error rate but higher processivity and this was evaluated against Phusion^®^ DNA polymerase. As indicated in Figure 2, DNA samples obtained using the three DNA preparation protocols were then used for amplification using either a hot-start FIREPol^®^ Taq DNA polymerase (Solis BioDyne, Tartu, Estonia) or Phusion^®^ DNA polymerase (New England Biolabs, Ipswich, MA, USA). Cost estimates were calculated according to the quantity of reagents needed to process 1000 samples and the estimates were calculated for both the DNA preparation methods as well as PCR assays. The prices were obtained from recent purchases of the reagents to be used in this study by the International Centre of Insect Physiology and Ecology (icipe), Kenya.

### 2.2. Flea Sampling

Field sampling for flea larvae was performed in Msambweni sub-county, coastal Kenya. Flea larvae were obtained from soil samples that were collected from the floors of households with at least one person infected with *T. penetrans*. The Berlese–Tullgren extraction method [29] was used to extract the larvae. It is a method by which soil arthropods are forced by a temperature gradient to move downwards and are trapped by a collection container. This was followed by the screening of the arthropod collection under a Zeiss Stemi 508 stereo microscope (Carl Zeiss Suzhou Co., Ltd., Suzhou, China) (magnification 6.3×) to separate other soil arthropods from the suspected *T. penetrans* larvae. Reference DNA was obtained from adult *T. penetrans* collected during previous studies [30] and from insectary-reared *C. felis* larvae maintained by artificial feeding at the Institute for Parasitology and Tropical Veterinary Medicine.

### 2.3. DNA Preparation Methods

#### 2.3.1. NucleoSpin^®^ Soil DNA Isolation Protocol

A NucleoSpin^®^ Soil protocol for the purification of DNA from soil and sediments was used (Macherey-Nagel, Düren, Germany). Individual larvae were homogenized using beads contained in the kit and a beating device (SpeedMill, Jena Bioscience, Jena, Germany). The isolation followed the manufacturer’s protocol and contained an inhibitor removal step optimized for soil samples. The final DNA pellets were eluted with 50 µL of elution buffer.

#### 2.3.2. Ammonium Acetate DNA Protocol

Larvae were transferred individually to 50 µL of tissue lysis buffer (10 mM TrisCl, pH 8.0, 0.5% sodium dodecyl sulphate, 5 mM EDTA) in a 1.5 mL microcentrifuge tube and crushed using a bleach-treated pestle. Another 100 µL of tissue lysis buffer was then added, before 60 µg of proteinase K was added. Samples were then incubated at 65 °C for 3 h. After incubation, 100 µL of 7.5 M ammonium acetate was added and the samples were mixed by rigorous shaking, before the samples were placed on ice for 15 min, followed by centrifugation at 14,500× *g* at 4 °C for 15 min. The supernatant was then transferred to a fresh 1.5 mL microcentrifuge tube that contained 150 µL of ice-cold 2-propanol, which was lightly shaken, placed on ice water for 30 min and then centrifuged at 14,500× *g* at 4 °C for 30 min. The resulting supernatant was removed and the pellet was washed with 150 µL of ice-cold 70% ethanol. Pellets were air-dried overnight. The DNA was finally dissolved in 50 µL of DNase-free water and stored at −20 °C until further use.

#### 2.3.3. Crude Flea Lysate Protocol

Larvae were transferred into individual microcentrifuge tubes that contained 50 µL of PCR-grade water and the tubes were placed in a water bath at 95 °C for 5 min. The larvae were then crushed using bleach-treated pestles and incubated at 95 °C for 5 min, after which they were left to cool down at room temperature (28 °C), before being stored at −20 °C until further use.

### 2.4. PCR Conditions

#### 2.4.1. PCR Primers

Two different primer pairs were used (Table 1). The *T. penetrans*-specific (TPS) primer pair amplifies an approximate 278 bp fragment of the *T. penetrans* internal transcribed spacer-2 (ITS-2) and is expected to be specific for *T. penetrans,* according to the manual comparison of ITS-2 sequences of *T. penetrans* and the closely related species *T. trimamillata*. For this purpose, both ITS-2 regions were aligned by Blastn and primers were placed in those regions where a maximal mismatch between *T. penetrans* and *T. trimamillata* was observed. The cytochrome oxidase 2 (*cox2*) primer pair has previously been used to amplify a partial mitochondrial DNA fragment from many species of Siphonaptera [31,32], including *T. penetrans* and *T. trimamillata* [33].

#### 2.4.2. GoTaq^®^ DNA Polymerase Conventional PCR Protocol

This PCR was only used in preliminary experiments in the beginning of the study and is not included in the scheme in Figure 2. PCRs were conducted in a final volume of 10 µL of 1× Green GoTaq^®^ Flexi Buffer (Promega Corporation, Madison, WI, USA), that contained 0.5 µM of each *cox2* primer pair (Table 1), 1.25 U of GoTaq^®^ Hot Start Polymerase (Promega Corporation, Madison, WI, USA), 2 mM of MgCl_2_ (Promega) and 0.2 mM of dNTPs (New England BioLabs, Ipswich, MA, USA).

A BIO-RAD T100™ Thermal Cycler (Marnes-la-Coquette, France) was used for the conventional PCR. After an initial denaturation at 95 °C for 30 s, 35 cycles at 95 °C for 10 s, at 53 °C annealing temperature for 30 s, and an extension at 72 °C for 45 s were performed before a final extension at 72 °C for 7 min.

The resulting PCR products were separated on 2% agarose gels that contained Safe View Classic DNA dye (Applied Biological Materials Inc., Richmond, BC, Canada), and the results were documented under UV light using the Syngene InGenius LHR2 Gel Imaging System (Scientific Laboratory Supplies Limited, Nottingham, UK). A 100 bp Hyperladder (610 ng) (Bioline Reagents Limited, London, UK) was used as a molecular weight marker.

*T. penetrans* genomic DNA from an adult, neosomic flea sample collected during a previous study [30] was included as a reference positive control and DNase-free water as a no template control (NTC).

#### 2.4.3. FIREPol^®^ Taq DNA Polymerase Real-Time PCR Protocol

PCRs were conducted in a final volume of 10 µL that contained 2 µL of template DNA, 0.5 µM of each primer pair (Table 1) and 1× Hot FIREpol^®^ EvaGreen^®^ (Solis BioDyne, Tartu, Estonia) for real-time PCR. Samples were initially denatured at 95 °C for 5 min, followed by 35 cycles at 95 °C for 10 s, annealing at the primer pair/method-specific annealing temperature (Table 1) for 30 s, and extension at 72 °C for 45 s, before a final extension at 72 °C for 7 min was conducted.

Real-time PCR analyses were conducted in a Mic qPCR cycler (Bio Molecular Systems, Upper Coomera, Australia). Fluorescence was measured during the extension period. Melting curves were obtained by heating the samples from 72 °C to 95 °C using a 0.1 °C/s ramp. Melting curves were plotted as relative fluorescence units vs. temperature and as the first derivative (−dF/dϑ) of the melting curve.

For all the PCR assays, *T. penetrans* genomic DNA from an adult, neosomic flea sample was included as a reference positive control and DNase-free water as a no template control (NTC). Additionally, for all the ITS-2 PCR assays, DNA from cat flea larvae was included as a negative control.

#### 2.4.4. Phusion^®^ HF DNA Polymerase Real-Time PCR Protocol

PCRs were conducted in a final volume of 10 µL 1 × HF buffer that contained 2 µL of template DNA, 0.5 M of each primer pair (Table 1), 0.2 U of Phusion^®^ High-Fidelity DNA Polymerase (New England BioLabs, Ipswich, MA, USA) and 0.5 mM of dNTPs (New England BioLabs, Ipswich, MA, USA). EvaGreen dye (Jena Bioscience, Jena, Germany) was added to a final concentration of 0.5 µM.

After initial denaturation at 98 °C for 30 s, 30 cycles at 98 °C for 10 s, annealing at the primer pair/method-specific annealing temperature for 30 s, and an extension at 72 °C for 45 s were performed, before a final extension at 72 °C for 7 min.

Positive and negative controls were used and real-time PCR analysis was performed as detailed above (Section 2.4.3).

### 2.5. Evaluation of the Specificity of the Tunga penetrans Partial ITS-2 PCR

The *T. penetrans* partial ITS-2 PCR was designed to discriminate *T. penetrans* from other flea species. In order to evaluate specificity, the PCR assays were applied to different template DNAs. Since no specimens of the most closely related flea species *T. trimamillata* were available in the present study, the sequence of the published ITS-2 was artificially synthesized and cloned in the p-SK-A plasmid vector (StrataClone PCR Cloning Kit, Agilent Technologies, Waldbronn, Germany). Plasmids were isolated from *Escherichia coli* cultures using a GenUP™ Plasmid Kit protocol (biotech rabbit, Berlin, Germany) for isolating high-copy-number plasmid DNA from 0.5–5 mL bacterial culture.

The Phusion^®^ HF DNA polymerase real-time PCR protocol was used and DNA isolated from cat flea larvae was also included in the analyses as a negative control for specificity.

### 2.6. PCR Efficacy Analyses for Real-Time PCRs

PCR efficacies were calculated for each sample based on changes in the normalized fluorescence vs. cycle number fitting of an exponential equation to the data using the LinRegPCR algorithm, as implemented in MIC PCR software version 2.8.10 (Bio Molecular Systems, Upper Coomera, Australia).

### 2.7. Statistical Analyses

Quantification cycles (C_q_) and PCR efficacies were compared between different DNA preparation methods after applying the sample PCR protocol using one-Way ANOVA in GraphPad 5.02. For comparison between different PCR protocols applied to the same set of DNA templates, paired *t*-tests were conducted. Success rates for PCRs between different protocols were compared using the mid-p exact test, as implemented in the tab2by2.test function in the R package epitools 0.5–10.1, using R version 4.1.1.

## 3. Results

### 3.1. Initial Comparison of Taq and Phusion^®^-Based PCR Protocols Using Conventional PCR

The soil kit method and the ammonium acetate method were initially compared using conventional PCRs based on amplification of a 730 bp fragment of the *cox2* gene. While amplification was successful for ten out of ten samples for both DNA preparation methods if Phusion^®^ polymerase was used, only six samples were amplified using GoTaq polymerase for the soil kit method and four samples for the ammonium acetate method. Given that the successful amplification using GoTaq^®^ is approximately 50% compared to 100% using Phusion^®^ polymerase, we decided to use the improved FIREPol^®^ Taq for better comparison with Phusion^®^.

### 3.2. Comparison of Different Combinations of DNA Preparation and Real-Time PCR Methods

The six different combinations of three DNA preparation methods and two different amplification protocols/polymerases were systematically evaluated using real-time PCRs that targeted a partial fragment of the ITS-2 region, which was designed to be *T. penetrans*-specific, and a partial cytochrome oxidase 2 fragment. Regarding the costs, there were strong differences between the protocols, with by far the highest costs being caused by the use of the S-kit for DNA isolation followed by the AmAcet method and only minimal costs were reported for DNA preparation with the CL protocol. For the polymerases, the FIREPol^®^ Taq was slightly more expensive than the Phusion^®^ polymerase. Thus, the combination of the S-kit with FIREPol^®^ was by far the most expensive protocol (USD 5207/1000 samples), while the combination of CL with Phusion^®^ polymerase (USD 260/1000 samples) was almost 20-times cheaper.

#### 3.2.1. Comparison of Combinations of DNA Preparation Methods and PCR Enzymes Based on a Partial Internal Transcribed Spacer 2 PCR

**Figure 3:**
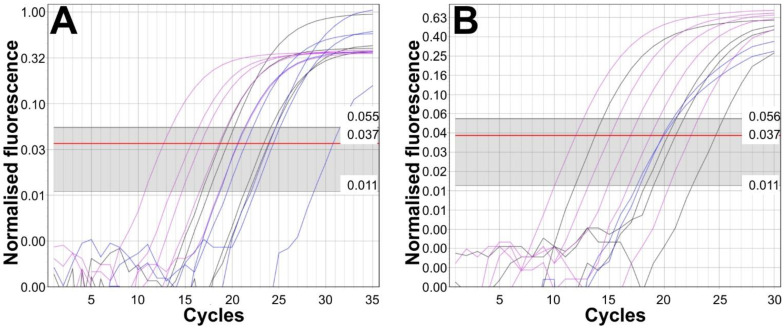
Representative amplification plots for the *Tunga penetrans* using FIREPol^®^ Taq (**A**) and Phusion^®^ (**B**) polymerases. The different DNA preparation methods are color-coded, including pink for AmAcet, blue for S-kit, and black for CL.

Representative amplification plots for the TPS ITS-2 PCR using different DNA preparation methods are shown for FIREPol^®^ Taq (Figure 3A) and Phusion^®^ polymerases (Figure 3B). For both polymerases, successful amplification was achieved in more than 80% of the samples (Table 2).

**Figure 4:**
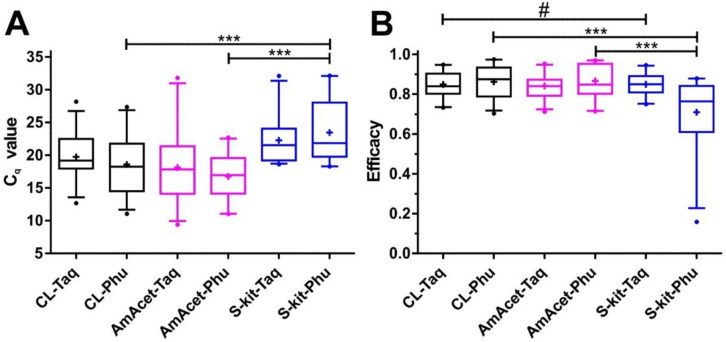
Comparison of the cycles of quantification (C_q_ value) (**A**) and PCR efficacies (**B**) between different DNA preparation methods and amplification protocols for a *Tunga penetrans* partial ITS-2 PCR. Template DNA was obtained (i) as a crude lysate (CL) by simple boiling of mechanically cracked larvae, (ii) by proteinase K digestion followed by precipitation with ammonium acetate (AmAcet) or (iii) using a soil DNA isolation kit (S-kit). Amplification was performed as real-time PCR using either the FIREPol^®^ Taq (Taq) or the Phusion^®^ (Phu) DNA polymerase protocols. Boxplots show medians with interquartile ranges and whiskers represent 5 and 95% quantiles. Outliers are indicated by dots. The mean of all values is shown as a cross. Paired *t*-tests for the same template DNA using either Taq or Phusion^®^ polymerase protocols did not reveal any significant differences. Comparison between different DNA preparation methods using the same amplification protocol were conducted using one-way ANOVAs. Hashtags were used to indicate differences between the DNA preparation methods for the Taq polymerase protocol, while asteriks indicate differences between the preparation methods for the Phusion^®^ protocol. #, *p* < 0.05; ***, *p* < 0.001.

There were no significant differences in the C_q_ values for the same template when the FIREPol^®^ Taq and the Phusion^®^ PCR protocols were compared (Figure 4A). However, the C_q_ values were higher for the S-kit DNA isolation method when Phusion^®^ polymerase was used. These differences were significant for both comparisons with the CL and AmAcet preparation protocols. For the FIREPol^®^ Taq polymerase, the S-kit DNA isolation method also had the highest median and mean C_q_ values and significant differences were observed, when compared with the CL and AmAcet protocols (Figure 4A).

In Figure 4B, PCR efficacies, as calculated by the LinRegPCR algorithm from the individual amplification plots, are shown. The PCR efficacies were very similar between the different methods. No significant effect of the DNA polymerase was observed. Comparisons of the DNA preparation protocol revealed significantly lower efficacies for the S-kit if used in combination with the Phusion^®^ amplification protocol. However, this was largely explainable by three replicates with very low efficacies (below 0.5) in the data set. For FIREPol^®^ Taq polymerase, no significant differences between the DNA preparation methods were detected.

#### 3.2.2. Comparison of Combinations of DNA Preparation Methods and PCR Enzymes Using cox2-Specific PCR

Using the same approach (and the same set of template DNAs) as for the partial ITS-2 PCR, the cox2 PCR was used to evaluate DNA preparation and amplification protocols. In this case, it was assumed that a PCR with a larger amplification product (278 bp vs. 730 bp) will show more pronounced differences between different protocols.

Table 2 shows a few of the significant differences in the success rate that were not observed for the partial ITS-2 PCR. For the AmAcet DNA preparation method, the FIREPol^®^ Taq protocol was significantly more frequently successful than the Phusion^®^ protocol. Moreover, when the FIREPol^®^ Taq protocol was used, AmAcet showed a higher frequency of PCR reactions with a positive amplification than the S-kit and the CL approach. Such differences were not observed for the Phusion^®^ polymerase (Table 2).

**Figure 5:**
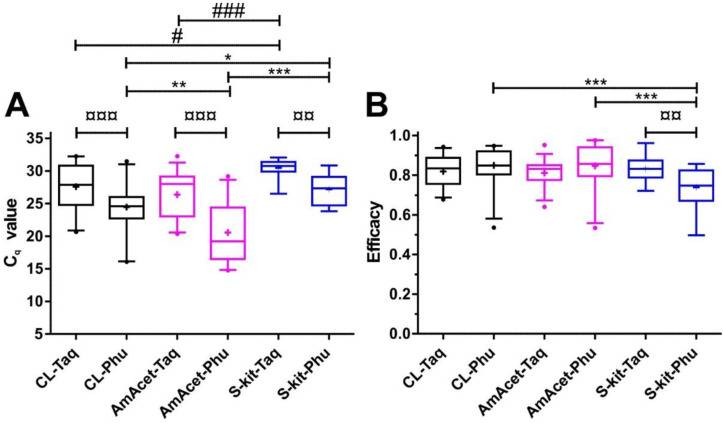
Comparison of the cycles of quantification (C_q_ value) (**A**) and PCR efficacies (**B**) between different DNA preparation methods and amplification protocols for a cox2 partial PCR. Template DNA was obtained as a crude lysate (CL) by simple boiling of mechanically cracked larvae and by proteinase K digestion, followed by precipitation with ammonium acetate (AmAcet) or using a soil DNA isolation kit (S-kit). Amplification was performed as a real-time PCR using either the FIREPol^®^ Taq (Taq) or the Phusion^®^ (Phu) DNA polymerase protocols. Boxplots show medians with interquartile ranges and whiskers represent 5 and 95% quantiles. Outliers are indicated by dots. The mean of all the values is shown as a cross. Paired *t*-tests for the same template DNA using either Taq or Phusion^®^ polymerase protocols did not reveal any significant differences. Comparisons between different DNA preparation methods using the same amplification protocol were conducted using one-way ANOVAs. # is used to indicate differences between the DNA preparation methods for the Taq polymerase protocol, while * indicates differences between the preparation methods for the Phusion^®^ protocol. ¤ is used to indicate differences between FIREPol^®^ Taq and Phusion^®^ in paired analyses. *, #, *p* < 0.05; ¤¤, **, *p* < 0.01; ¤¤¤, ***, ###, *p* < 0.001.

Regarding the C_q_ values, Phusion^®^ polymerase produced significantly lower C_q_ values than FIREPol^®^ Taq for all three DNA preparation protocols (Figure 5A). Comparison of the DNA preparation methods based on FIREPol^®^ Taq polymerase showed lower C_q_ values for the CL and AmAcet methods, when compared with the S-kit. For Phusion^®^ polymerase, all comparisons between the methods were significant, with the lowest C_q_ values for AmAcet, followed by the CL and the S-kit protocol (Figure 5A). By analyzing the PCR efficacy data, only the combination of the S-kit with the Phusion^®^ amplification protocol revealed significant differences compared to the other protocol combinations (Figure 5B). The S-kit/Phusion^®^ combination showed significantly lower efficacies than the S-kit/FIREPol^®^ Taq protocol and also than the CL and AmAcet methods in combination with Phusion^®^ polymerase (Figure 4B).

### 3.3. Specificity of Tunga penetrans Partial ITS 2 PCR

Tunga penetrans and T. trimamillata plasmids based on their published ITS-2 gene sequences were used to evaluate the specificity of the T. penetrans-specific primer pair using the Phusion^®^ DNA polymerase real-time PCR protocol. Independent of the amount of template DNA (10^1^–10^5^ copies per reaction), amplification was observed for all three replicates with the T. penetrans plasmid as a template, while all replicates with T. trimamillata ITS-2 used as a template were negative. Moreover, no cross reaction with cat flea DNA prepared with the CL protocol was observed (Table 3).

## 4. Discussion

The aim of this study was to establish DNA preparation and PCR protocols that allow low-cost, high-throughput molecular processing of large numbers of *Tunga* off-host stages in preparation for extensive ecological and epidemiological tungiasis risk factor studies. Six protocols, including all combinations of three DNA preparation methods with two PCR enzymes, were compared to identify a reliable and low-cost method for the identification of *T. penetrans,* with a particular focus on off-host stages such as larvae, for which no morphological keys are currently available. The *T. penetrans*-specific partial ITS-2 primer pair is the first pair designed to discriminate *T. penetrans* from other flea species, including *T. trimamillata,* and can, in the future, be used to identify juvenile off-host and adult on-host stages of *T. penetrans* in field samples. This approach can then be used to document the morphology of all stages of the flea species.

Following DNA preparation, PCR assays that targeted the *cox2* gene were initially performed using a conventional hot-start Taq polymerase (GoTaq) and Phusion^®^ polymerases. However, GoTaq, the cheapest polymerase used here, was not further evaluated due to an unsatisfactory success rate compared to Phusion^®^. Instead, FIREPol^®^ Taq and Phusion^®^ were compared in all of the subsequent experiments. Both of these polymerases have a higher processivity than conventional Taq and were chosen to compensate for the potential presence of PCR inhibitors in soil samples. An alternative approach is the removal of such inhibitors during the DNA preparation process using commercially available kits, which are optimized to extract DNA from microorganisms in soil samples. Due to an additional purification step, they are considerably more expensive than the DNA extraction kits used for tissue samples.

Among the three DNA preparation methods, the S-kit turned out to be the most expensive and showed the poorest results in terms of success rate for PCR, highest C_q_ values and lowest PCR efficacy. Differences between the two low-cost methods—AmAcet and CL—were negligible, although there was a tendency for lower C_q_ values and higher PCR success rates for the AmAcet approach compared with the crude lysate prepared by mechanical disruption and boiling in the CL protocol. However, the CL protocol has the advantage that it only requires a water bath or heat block and no further laboratory equipment, such as a centrifuge. It also requires only minimal handling of samples, which reduces the risk of the contamination of samples. The poor performance of the S-kit in comparison to the other methods was unexpected. A likely explanation might be the very small amount of starting material. Soil DNA kits are optimized for a defined amount of soil, which in this case was 500 mg; however, *T. penetrans* larvae are less than half of the size of a *C. felis* larva and even unfed adult *C. felis* have a weight below 0.5 mg [34]. With such a small amount of starting material, the S-kit protocol might result in a sub-optimal DNA yield.

Differences between the types of polymerases were also small. If significant differences in the amplification efficiency were found, they were most often observed for the larger *cox2* PCR product. On the one hand, the number of successful sample amplifications of the *cox2* PCR was significantly higher for FIREPol^®^ Taq than for Phusion^®^ when the AmAcet protocol was used for DNA preparation, as compared to the other two DNA preparation methods. On the other hand, the C_q_ values were significantly lower for Phusion^®^ than for FIREPol^®^ Taq with all three DNA preparation methods. Since this was not accompanied by higher PCR efficacies, as determined using LinRegPCR, the significantly lower C_q_ values in the paired data analyses using the same template DNA suggests that other differences in the PCR protocols contributed to this effect. One possible explanation is the brighter fluorescence signal of double-stranded DNA in the Phusion^®^-based assay. Even though both assays use EvaGreen as double-stranded DNA-specific dye, the differences in EvaGreen concentration cannot be excluded as a cause for the different results, since the EvaGreen concentration is not provided by the supplier in the product information of the FIREPol^®^ EvaGreen qPCR Supermix. Another important difference between both PCR reaction mixtures is the presence of dUTP in the FIREPol^®^ EvaGreen qPCR Supermix, which is known to be incompatible with some PCR enzymes. However, since no significant differences were observed in the PCR efficacy in the exponential amplification phase, the presence of dUTP is an unlikely explanation.

The use of the soil kit leads to costs that are approximately six-fold higher than that of the AmAcet approach and are twenty-fold higher than that of the CL approach. Both AmAcet and CL combined with either of the polymerases deliver acceptable results and the decision on the method can be based on the price, which clearly favors the combination of CL with Phusion^®^ polymerase.

The choice of primer pairs for the present study was guided by two different considerations. The *cox2* primer pair has been frequently used in studies on the phylogeny of Siphonaptera and is, therefore, well known to amplify partial mitochondrial DNA fragments from many flea species [31,32]. This also means that there is a considerable number of flea *cox2* sequences available in GenBank and this PCR can, therefore, be used in future projects to identify larvae from species that were negative in the *T. penetrans*-specific PCR, leading to an improvement of our knowledge regarding the specificity of the partial *T. penetrans* ITS-2 PCR over time. The *T. penetrans*-specific PCR can be used in ongoing and future projects to rapidly identify larvae collected in households or stables to identify sites of *T. penetrans* development and transmission. It can also be used to replace morphological identification of adult *Tunga* spp.; however, morphological identification is possible for *Tunga* spp. using a published key [34].

In South America, there are three confirmed synanthropic *Tunga* species that infect humans, companion animals and/or livestock, i.e., *T. penetrans* , *T. trimamillata* (both zoonotic) and *Tunga hexalobulata* (only known to infect cattle so far) [2,35], in addition to *Tunga caecata*, which infects synantropic rats [3]. In this context, further evaluation of the specificity of the *T. penetrans*-specific PCR is required. In Africa, only *T. penetrans* is endemic and it will be sufficient to confirm the specificity of the PCR by sequencing the PCR product for a small subset of positive samples to confirm its identity in future field studies.

In conclusion, the present study has evaluated a set of DNA preparation/PCR protocols and identified low-cost approaches to identify flea larvae from soil samples. The approximately 20-fold decrease in costs compared to the use of a soil DNA isolation kit is highly relevant for resource-poor settings and the developed low-cost protocols will allow us to screen much higher numbers of samples collected in field studies. While *T. penetrans* can be directly detected using a species-specific PCR or any flea larvae by a *cox2* PCR, followed by sequencing, applying these PCRs in future field studies will allow us to further characterize their sensitivity and specificity. In the future, the same approach can also be adapted to be used for other arthropods from PCR-inhibitor-rich matrices, such as soil or feces.

## Figures and Tables

**Table 1:** Primer information. Target gene and primer sequences for both forward and reverse primers.

Target Gene	Primer Name	Primer Sequence (5′->3′)	Size (^a^bp)	Annealing Temperatures (°C)	Reference
*Tunga penetrans* ITS-2	TPS-F	TGCTCGACCCGGTGACGGGA	278	FIREPol^®^ Taq 65 Phusion^®^ 69	This study
	TPS-R	CGCGCAAAGCGTGGAGGTTTCG			
*Cox2*	F-Leu	TCTAATATGGCAGATTAGTGC	730	GoTaq 53FIREPol^®^ Taq 53 Phusion^®^ 53	[32]
	R-Lys	GAGACCAGTACTTGCTTTCAGTCATC			

^a^bp, base pairs.

**Table 2:** Success rate for different PCRs and DNA preparation methods based on 30 replicates.

	FIREPol^®^ Taq				Phusion^®^			*p* Value		
PCR	DNA Preparation	n	% pos.	95% CI	n	% pos.	95% CI	FIREPol^®^ vs. Phusion^® a^	FIREPol ^b^	Phusion^® b^
*Tunga penetrans* partial ITS-2										
	S-kit	28	93.3	78.7–98.1	24	80.0	62.7–90.5	0.153	1	0.057
	AmAcet.	28	93.3	78.7–98.1	29	96.7	83.2–99.4	0.619	0.433	0.753
	CL	26	86.7	70.3–94.7	25	83.3	66.4–92.7	0.736	0.433	0.109
*Cox2*										
	S-kit	17	56.7	39.1–72.6	17	56.7	39.1–72.6	1	<0.001	0.191
	AmAcet.	30	100	88.7–100	22	73.3	55.6–85.8	0.002	0.112	0.112
	CL	23	76.7	59.1–88.2	23	76.7	59.1–88.2	1	0.005	0.776

n, number of successful PCRs; 95% CI, 95% confidence interval. ^a^ Comparison of results for FIREPol^®^ Taq and Phusion^®^ polymerases conducted on the same set of samples. ^b^ Comparison between different DNA preparation protocols using the same polymerase and mid-p-exact tests. *p* values are given from top to bottom for the comparisons of S-kit vs. AmAcet, S-kit vs. CL and AmAcet vs. CL.

**Table 3:** Specificity of the *Tunga-penetrans*-specific PCR. Different amounts of plasmids containing the ITS-2 region of *T. penetrans* and *Tunga trimamillata* and genomic DNA from *Ctenocephalides felis* larvae were used as template for the *T. penetrans*-specific real-time PCR.

Target Quantity (Copy Numbers)	1 × 10^5, a^	1 × 10^3, a^	1 × 10^1, a^
	C_q_ Value (Mean (range))	C_q_ Value (Mean (range))	C_q_ Value (Mean (range))
Target Species			
*T. penetrans*	22.49 (2.946)	25.81 (5.239)	28.46 (3.198)
*T. trimamillata*	n.a.	n.a.	n.a.
*C. felis* (genomic DNA) ^b^	n.a.	n.a.	n.a.

n.a., not available. ^a^ n = 3. ^b^ copy number unknown.

## Data Availability

Data are contained within the article.
